# Improved Electrochemical Performance of Surface Coated LiNi_0.80_Co_0.15_Al_0.05_O_2_ With Polypyrrole

**DOI:** 10.3389/fchem.2018.00648

**Published:** 2019-01-09

**Authors:** Zhaoyong Chen, Kaifeng Cao, Huali Zhu, Xiaolong Gong, Qiming Liu, Junfei Duan, Lingjun Li

**Affiliations:** ^1^College of Materials Science and Engineering, Changsha University of Science and Technology, Changsha, China; ^2^College of Physics and Electronic Science, Changsha University of Science and Technology, Changsha, China; ^3^Department of Chemistry, University of New Hampshire, Durham, NH, United States

**Keywords:** nickel-rich layered oxide, polypyrrole coating, cathode materials, conductive polymer, lithium-ion batteries

## Abstract

Nickel-rich ternary layered oxide (LiNi_0._80Co_0.15_Al_0.05_O_2_, LNCA) cathodes are favored in many fields such as electric vehicles due to its high specific capacity, low cost, and stable structure. However, LNCA cathode material still has the disadvantages of low initial coulombic efficiency, rate capability and poor cycle performance, which greatly restricts its commercial application. To overcome this barrier, a polypyrrole (PPy) layer with high electrical conductivity is designed to coat on the surface of LNCA cathode material. PPy coating layer on the surface of LNCA successfully is realized by means of liquid-phase chemical oxidation polymerization method, and which has been verified by the scanning electron microscopy (SEM), transmission electron microscope (TEM) and fourier transform infrared spectroscopy (FTIR). PPy-coated LNCA (PL-2) exhibits satisfactory electrochemical performances including high reversible capacity and excellent rate capability. Furthermore, the capability is superior to pristine LNCA. So, it provides a new structure of conductive polymer modified cathode materials with good property through a mild modification method.

## Introduction

Rechargeable lithium ion batteries (LIBs) (Zheng et al., 2018; Zhang et al., 2018a,b) have been applied successfully to electric vehicles (EVs) (Xu et al., 2015) and hybrid electric vehicles (HEVs) (Goodenough and Park, 2013; Choi and Aurbach, 2016; Chen et al., 2017), which brings a great convenience and reduces exhaust emissions. However, the unsatisfied performance of cathode materials in LIBs, limits its extensive application in EVs and HEVs in the future, including low energy density, poor cycling performance et al. (Thackeray et al., 2012; Goodenough and Kim, 2014) Therefore, it is expected that the cycling performance and rate capability would be greatly improved.

LiNi_0.80_Co_0.15_Al_0.05_O_2_ (LNCA) produced by substituting Co and Al for Ni in LiNiO_2_ has been considered as a promising cathode material because of it has higher capacity and lower price than LiCoO_2_ (Delmas et al., 1993; Levi et al., 1999; Cao et al., 2004). Nonetheless, the LNCA undergoes drastic capacity fading and poor cyclability due to the increase in interfacial resistance on the surface of cathode (Amine et al., 2001; Itou and Ukyo, 2005), micro-cracks generated in the particles (Watanabe et al., 2014a,b), and formation of NiO-like rock-salt domains (Abraham et al., 2002, 2003; Hayashi et al., 2014). Currently, an effective method to enhance the electrochemical properties of LNCA is to modify its surface with some stable coating layer like oxides(Cho and Cho, 2010; Cho et al., 2013; Lai et al., 2016; Dai et al., 2016a,b; Yan et al., 2017), phosphates(Kim and Cho, 2007; Lee et al., 2011; Huang et al., 2014), fluoride (Kim et al., 2008; Lee et al., 2013; Zhang et al., 2014; Liu et al., 2016), and other cathode material (Liu et al., 2012; Du et al., 2013; Lu et al., 2014; Zhao et al., 2017), etc. These coating layers can inhibit effectively side reactions between electrode and electrolyte. However, the coating materials impede the diffusion of lithium ions and electronic transmission in a degree.

Recently, conducting polymers have been investigated as additives to improve the electrode performance (Chen et al., 2013; Wu et al., 2013; Ramkumar and Minakshi, 2015, 2016a; Ramkumar and Sundaram, 2016b) because the polymer can provide a high electrical conductivity for the active materials. Nonetheless, polypyrrole (PPy) modified LNCA cathode material via liquid-phase chemical oxidation polymerization method has been not reported yet. In order to improve the conductivity and initial coulombic efficiency of LNCA, we have demonstrated that an ultrathin ppy layer can enhance the electrical conductivity of LNCA via a liquid-phase chemical oxidation polymerization method using ammonium persulphate as oxidant, the ppy layer is highly conductive and contributes to the fast electron transportation on the surface of LNCA particles. So, the ppy coated LNCA reaches a high electrochemical capacity of 206.6 mAh g^−1^ at 0.1 C with coulombic efficiency of 91% and the optimized sample maintains 195.1 mAh g^−1^ with 88.9% capacity retention after 100 cycles at a current density of 1 C with the voltage of 2.75–4.3 V vs. Li/Li^+^ at room temperature, because the PPy reduces the interfacial impedance of the cathode material and provides high electrochemical reaction kinetics, leading to a superior structural stability during lithiation and delithiation cycling.

## Experimental

### Material Synthesis

LiNi_0.80_Co_0.15_Al_0.05_O_2_ (LNCA) powder was prepared by mixing Ni_0.80_Co_0.15_Al_0.05_(OH)_2_ and LiOH·H_2_O (99%, Sinopharm Chemical Reagent) at molar ratio of 1:1.05 and firing at 450°C for 5 h, subsequently calcining at 750°C for 12 h in O_2_ flow. After sieving, powders with an average particle size of 10 μm were used for further experiments.

**Figure 1:**
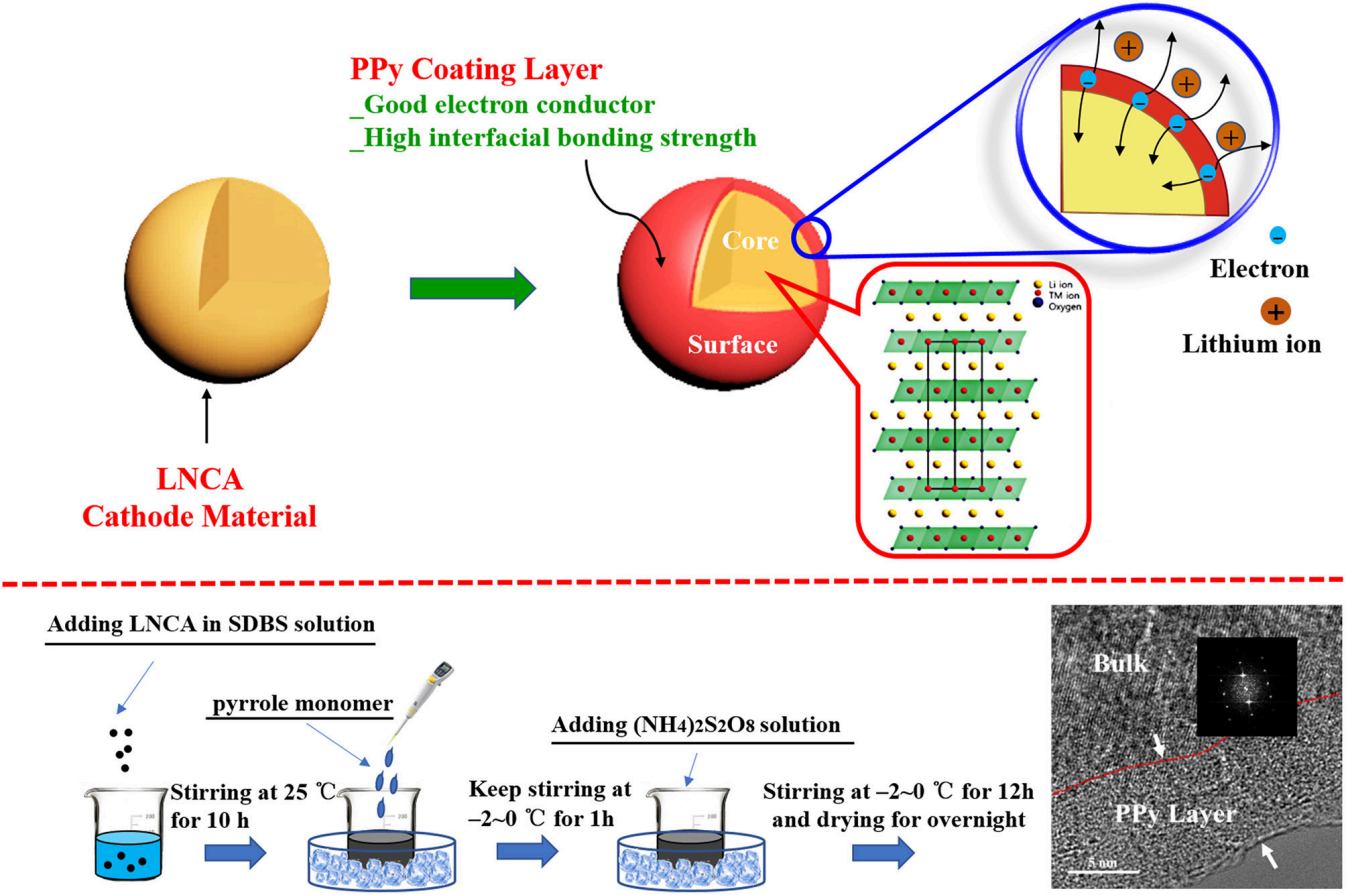
Schematic view of PPy coated LNCA architecture with high electrical conductivity and the synthetic process.

PPy-coated LiNi_0.80_Co_0.15_Al_0.05_O_2_ was prepared by wet chemical method in Figure 1 Desired amounts of pyrrole monomer (AR, 99%) and sodium dodecyl benzene sulfonate (SDBS) as doping agent and 2 g LiNi_0.80_Co_0.15_Al_0.05_O_2_ powder were ultrasonically dispersed in 100 ml deionized water. The aqueous solution of ammonium persulfate (AR, 99%) was slowly added as an oxidizing agent, and the concentration of ammonium persulfate was 0.06 M. The molar ratio of pyrrole monomer to oxidant and sodium dodecyl benzene sulfonate was 1:1, 1:1.5, respectively. The mixture was kept in ice-bath for 12 h under magnetically stirring and polymerized on the surface of the LNCA. The PPy coated samples were filtered, washed with deionized water and ethanol for several times, and finally dried under vacuum at 80°C for overnight. Finally, the PPy coated samples (PL-1, PL-2, and PL-5 corresponding to 1 wt, 2 wt, and 5 wt% PPy) were obtained.

### Material Characterization

Crystal structures of the prepared LiNi_0.80_Co_0.15_Al_0.05_O_2_ (LNCA) and PPy coated LNCA were characterized by X-ray powder diffraction (XRD) with Cu Kα radiation at 40 kV and 40 mA in the 2θ range from 10° to 90°. The morphology was characterized by field emission scanning electron microscopy (FESEM, FEI QUANTA 250). Transmission electron microscopy (TEM) and high-resolution transmission electron microscopy (HRTEM) were carried out on a JEOL JEM-2100 instrument.

### Electrode Preparation and Electrochemical Measurements

For electrochemical tests, the cathode materials were tested using a coin type cell (2025). The working electrode was prepared by pressing a powder mixture of the sample (PPy-NCA or pristine LNCA), Super P conductive carbon black and polyvinylidene difluoride (PVDF) in N-methyl-2-pyrrolidone (NMP) with a weight ratio of 80:10:10 and coated onto an aluminum foil and dried at 120°C in vacuum. Two electrode button cells (half cells) were assembled in an argon filled glove box using fresh lithium foil as the counter electrode, The electrolyte was 1 M LiPF_6_ dissolved in ethyl carbonate (EC) and dimethyl carbonate (DMC) (1:1 in volume) and the separator was Cellgard 2400 membrane. The charged / discharged measurements on a LAND battery-testing instrument within 2.75–4.3 V vs. Li/Li^+^ at 25°C. For cycling tests, the cells were cycled at the rate of 1 C (1 C rate is defined as 180 mAh g^−1^). The rate capability was measured at rates of 0.1 C, 0.2 C, 0.5 C, 1 C, 3 C, and 5 C. A cyclic voltammogram (CV) test was conducted between 2.75 and 4.3 V at a scanning rate of 0.1 mV s^−1^ using a CHI 660E electrochemical work station. The electrochemical impedance spectroscopy (EIS) of the cells was conducted using the CHI 660E electrochemical measurement system in a frequency range from 1 mHz to 100 kHz.

The electrodes after many cycles were prepared by disassembling coin cells fully discharged to 2.75 V and rinsed with dimethyl carbonate (DMC) several times in the glovebox, in order to investigate that the microstructure and morphology of cathode materials after long-term cycles.

## Results and Discussion

### Structural Characterization

**Figure 2:**
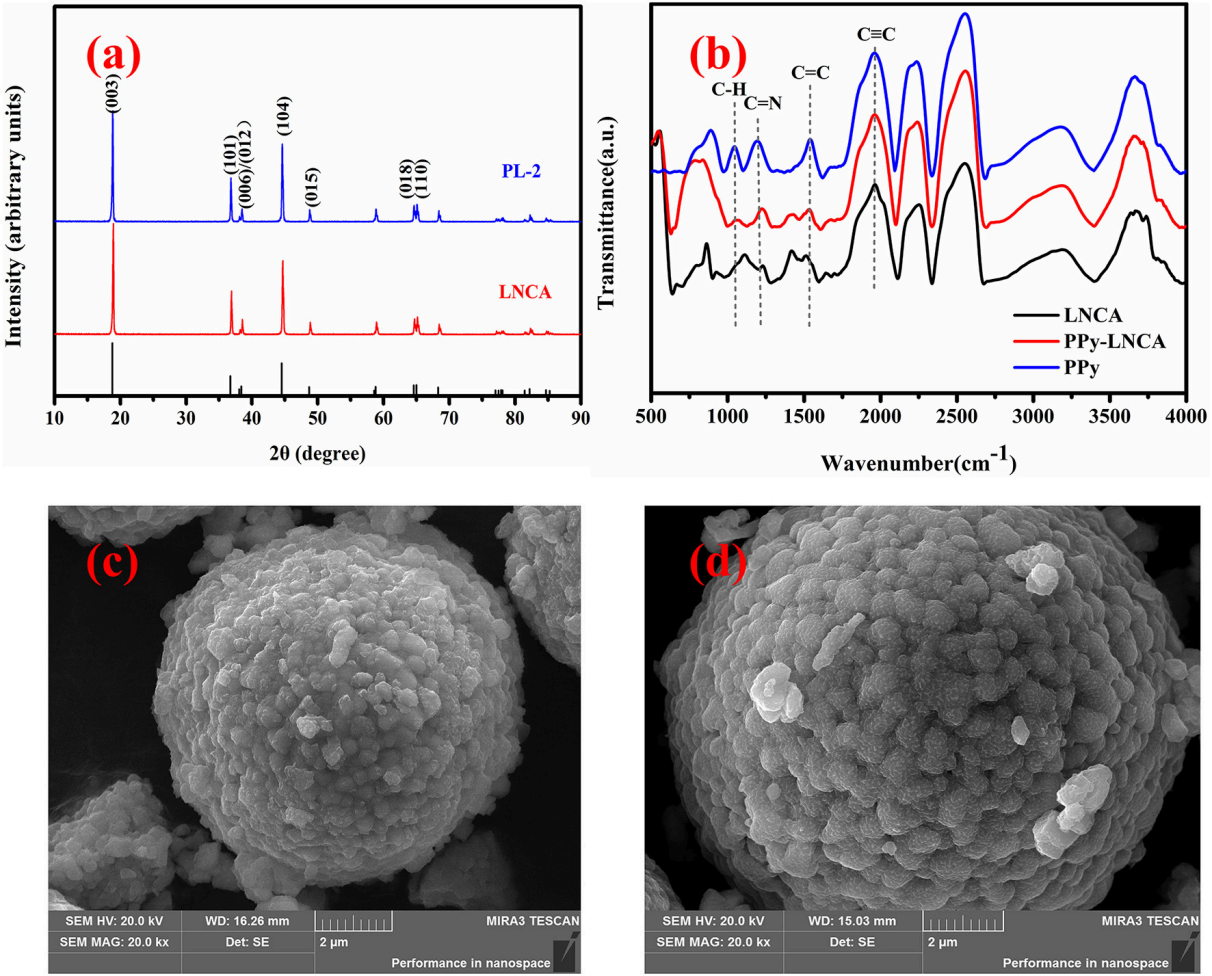
XRD and FTIR pattern and SEM images of pristine LNCA and PL-2 cathode material: **(a)** XRD pattern of pristine LNCA and PL-2 cathode material, **(b)** The FTIR spectra of pristine LNCA, PPy, and PPy-coated LNCA, **(c)** pristine LNCA morphology, **(d)** PL-2 samples exhibiting obscure fringes which are related to the coating of the ultrathin PPy layer.

Figure 2a shows the XRD pattern of pristine LNCA and PL-2 samples (PL-1 and PL-5 is shown Figure S1). The diffraction peaks of both samples are well indexed in terms of *R-3m* structure of hexagonal α-NaFeO_2_, which match well with those of the standard XRD patterns for (Li_0.99_ Ni_0.01_) (Ni_0.798_ Co_0.202_) O_2_ (PDF#: 87-1562). It should be noted that the pair reflections (006)/(102) and (018)/(110) are well-split for all samples, demonstrating the good hexagonal ordering and layered characteristics (Ohzuku et al., 1993). The intensity ratio of the (003) and (104) lines in the XRD patterns is more than 1.4 for all samples, indicating the suppressed cation mixing at a degree (Yang et al., 2014).

The Fourier transform infrared spectroscopy (FTIR) spectra of pristine LNCA, PPy, and PPy-coated LNCA are shown in Figure 2b. It is found that the PPy-coated LNCA has typical absorption peaks of PPy. The band at 1,541 cm^−1^ is due to aromatic C=C in PPy. C=N shows peaks around 1,193 cm^−1^. Obviously, the PPy-coated LNCA sample has a stronger absorption peak than pristine LNCA at the position of C=C and C=N spectra. The region of the C–H in-plane deformation vibration situated at 1,043 cm^−1^ are observed at the position of PPy-LNCA spectra(Blinova et al., 2007). The results demonstrate that the PPy is successfully coated onto the surface of LNCA particles.

The surface morphology of the pristine LNCA and PL-2 samples are shown in Figure 2. Apparently, the spherical morphology of the pristine LNCA is well-retained, and the estimated average particles diameter of the LNCA (Figure 2c) is around 8 μm. Furthermore, a fuzzy layer was obviously observed in the surface of LNCA in Figure 2d, which may be PPy films grown by liquid-phase chemical oxidation polymerization method. And it is in accordance with the above-mentioned FTIR results.

**Figure 3:**
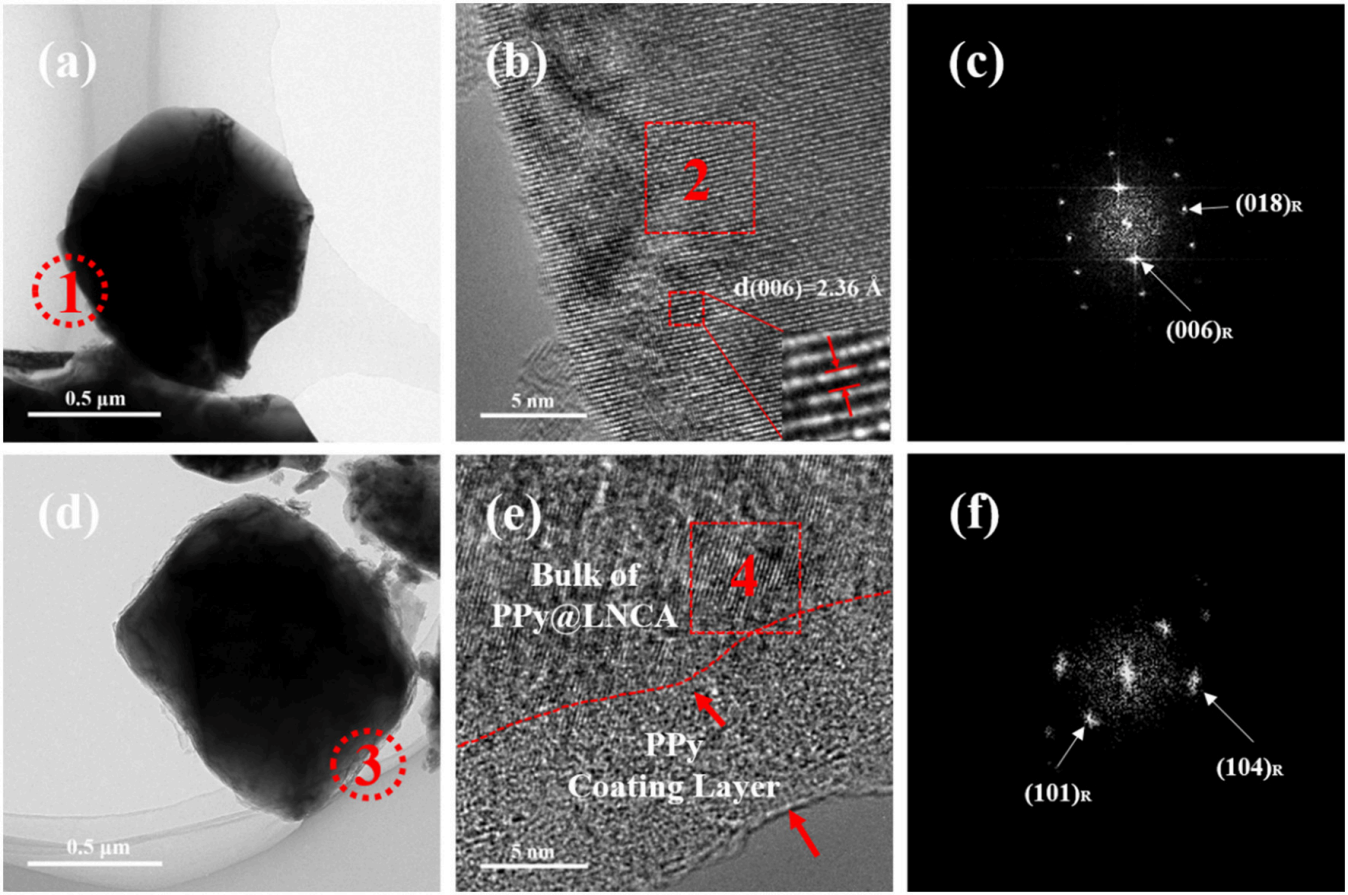
TEM images: **(a)**, **(d)**, HRTEM images: **(b)**, **(e)** of pristine LNCA, PL-2, respectively and corresponding FFT images(**c** and **f**) from the selected region.

The TEM (Figures 3a,d) and HRTEM (Figures 3b,e) images combined with the corresponding selected-area fast Fourier transform (FFT) pattern (Figures 3c,f) further confirm that the spherical morphology of the LNCA is composed of a well crystallized layered structure. As can be seen from Figures 3a,c, pristine LNCA particles exhibit a smooth surface and show a highly ordered lattice.

Bright and dark zones of a few nanometers wide are based on the inset of Figure 3b. The electron diffraction spots obtained after Fast Fourier Transform (FFT) were calculated and the crystal surface spacing was 0.236 nm, which was deduced to be the inter-planar spacing of the (006) plane of the layered structure. HRTEM images in Figure 3e shows that a nanoscale PPy film layer with a thickness of 10~12 nm is clearly seen on the surface of LNCA particles. It should be noted that the PPy layer is highly conductive, contributing a lot to the improvement of rate capacity of the LNCA cathode material. As depicted in Figure 3f, corresponding selected-area fast Fourier transform (FFT) pattern (in region 4 of Figure 3e) further confirm that the bulk of PL-2 still maintain the ordered layered structure. According to the results of SEM, TEM and HRTEM, it is concluded that a PPy nano layer is successfully coated on the surface of LNCA cathode materials.

### Electrochemical Performance

**Figure 4:**
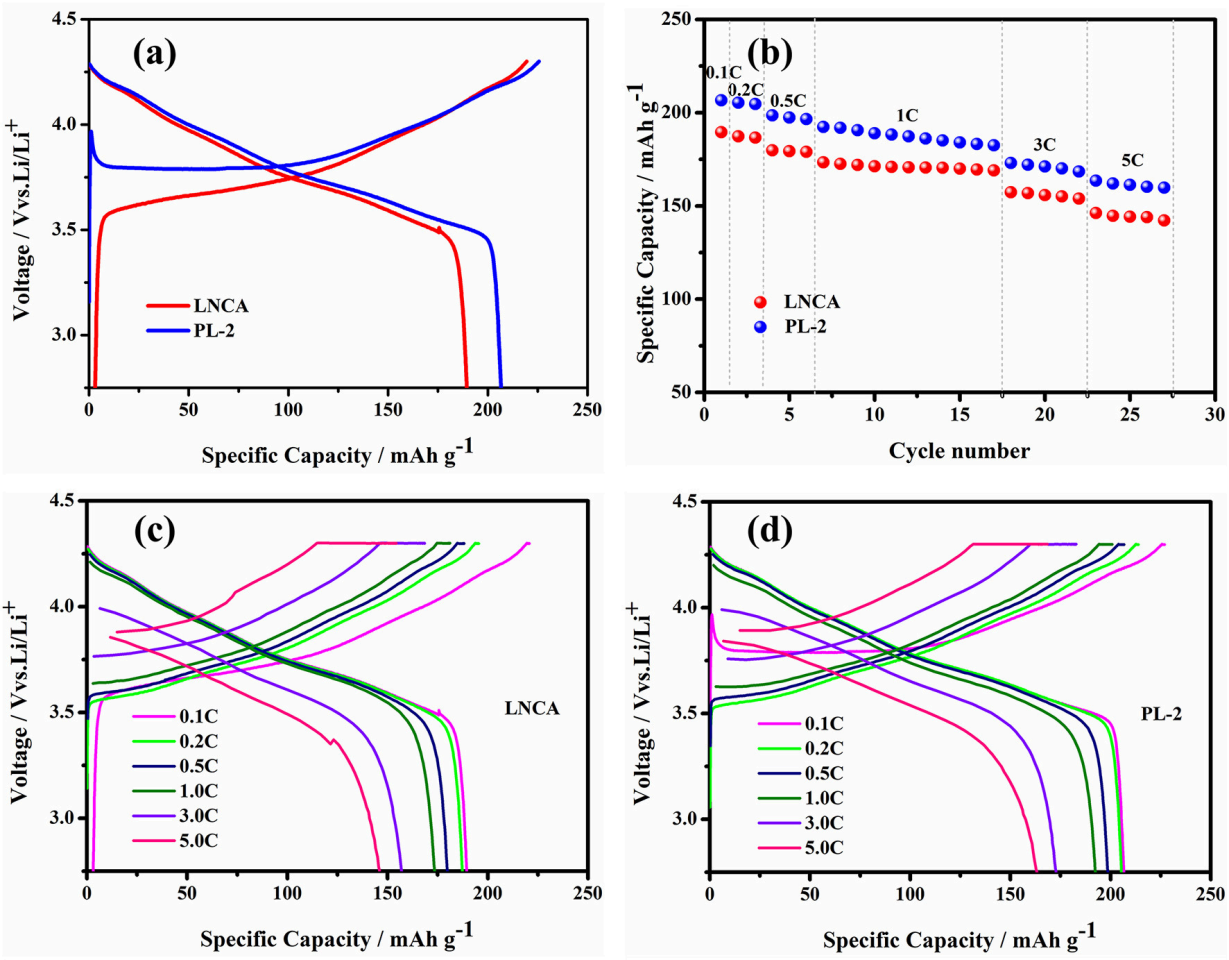
**(a)** Initial charge/discharge profiles at 0.1 C in the voltage range of 2.75–4.3 V, **(b)** rate performance, **(c,d)** initial charge/discharge profiles at various rates of pristine LNCA and PL-2.

The electrochemical performance of pristine LNCA cathode materials and PL-2 are evaluated and shown in Figure 4. The electrochemical performance of PL-1, PL-5 samples are presented in the ESI (Figures S2–S4). The initial charge/discharge profiles at 0.1 C rate are shown in Figure 4a. The pristine LNCA cathode materials deliver a discharge capacity of 189.5 mAh g^−1^ with an initial coulombic efficiency of 85.9%. However, for the PL-2 cathode materials, the discharge capacity approaches 206.6 mAh g^−1^, with an initial coulombic efficiency of 91%. The rate capability combined with corresponding charge/discharge profiles at various rates (Figures 4c,d) shows a superiority of PL-2, achieving higher rate capabilities than pristine LNCA. The voltage and discharge capacity of the cells gradually decrease with an increase of discharge current density due to the effect of ohmic polarization and interphase resistances caused by the side reaction on the interface between LNCA cathode and electrolytes. However, the higher discharge capacities of PL-2 are 163.5 mAh g^−1^ at 5 C, more than 146.2 mAh g^−1^ of pristine LNCA in Figure 4b. These test results indicate that PPy coating greatly reduces the electrode resistance of the cathode material and provides high electrochemical reaction kinetics, which ensured less discharge potential drop during high-rate cycling.

**Figure 5:**
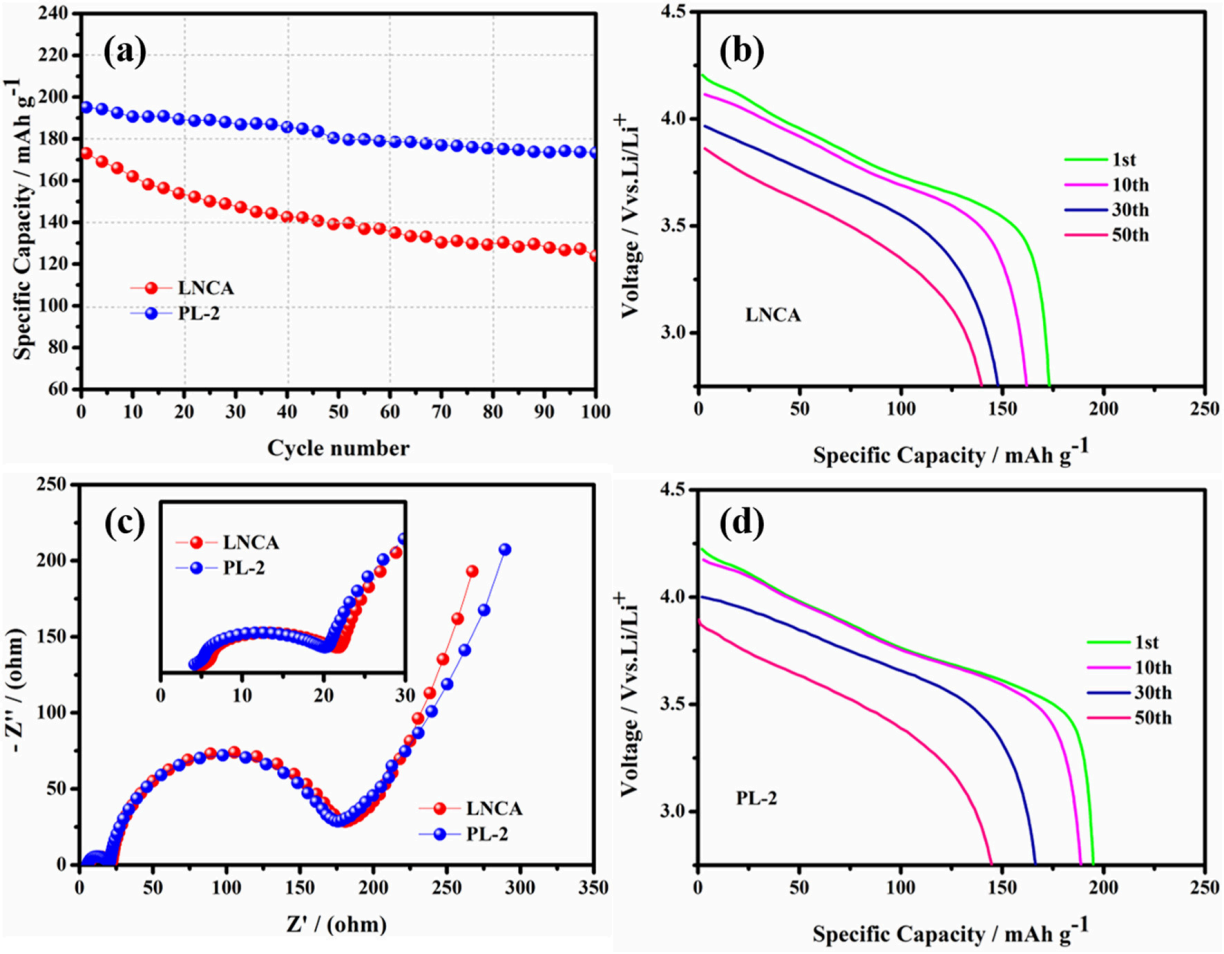
**(a)** Cycling performance at 1.0 C rate in the voltage range of 2.75–4.3 V, **(b,d)** initial discharge profiles evolution during cycling of pristine LNCA and PL-2. **(c)** Comparison of the electrochemical impedance spectra (inset is a magnified view of the high frequency region and equivalent circuit diagram) of the pristine and PL-2 charged to 4.3 V vs. Li/Li^+^.

Figure 5 shows the cycling performance and electrochemical impedance spectra of pristine LNCA and PL-2 samples. As observed, the discharge capacities of both samples decrease with the increasing of the cycle number. As can be seen from Figure 5a, the capacity retention of the PL-2 sample is 88.9% after the 100 th cycle at 1 C rate between 2.75 and 4.3 V, while the prestine LNCA material exhibits 71.6% capacity retention after 100 cycles. For the PL-2 sample, corresponding to the initial discharge profiles evolution during cycling of both samples in Figures 5b,d, the cycle performance is much improved. It is definitely demonstrated that the PL-2 show better structure stability than pristine LNCA. The poor cycling performances of the pristine LNCA can be attributed to its vigorous surface reactivity with electrolyte, where the harmful interfacial side reactions suppress lithium ion diffusion.

Electrochemical impedance spectroscopy (EIS) measurements are used to elucidate the beneficial effect of the PPy coating layer for LNCA material. As can be seen from Figure 5c, the depressed semicircle in the high frequency region is related to the surface film impedance of the active cathode material (Wu et al., 2017). A magnified view of the high frequency region curve and equivalent circuit diagram are shown in the inset. The Li^+^ ionmigration resistance (Rsei) and the charge transfer resistance (Rct) of different samples is listed in Table 1. Obviously, it is easy to conclude that PL-2 material has smaller electrochemical impedance compared to the pristine sample before cycling. The mid frequency region responds to the charge transfer impedance. It indicates that PPy coating reduces the charge transfer resistance of the material due to the high electrical conductivity of the PL-2 sample, which facilitates transfer of electrons from the conductive agent and current collector to the cathode material and the electrode materials are transfer to each other.

**Table 1:** The Li^+^ ion migration resistance (Rsei) and the charge transfer resistance (Rct) of different samples.

**samples**	**R_**sei**_(Ω)**	**R_**ct**_(Ω)**
LNCA	13.88	128.7
PL-2	12.94	123.2

**Figure 6:**
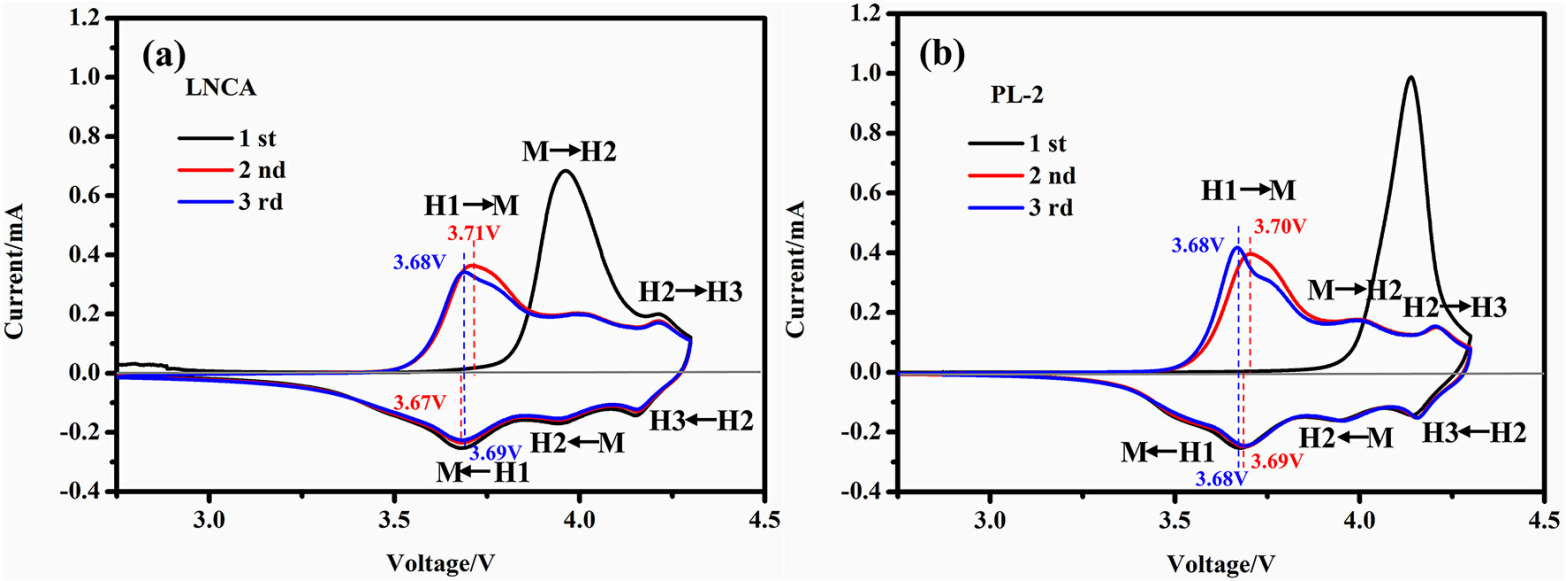
**(a,b)** the cyclic voltammetry (CV) curves of LNCA and PL-2 between 2.75 and 4.3 V vs. Li/Li^+^ at a scan rate of 0.1 mV s^−1^.

To investigate the degree of the electrochemical reversibility, the cyclic voltammetry (CV) curves of LNCA and PL-2 between 2.75 and 4.3 V vs. Li/Li^+^ at a scan rate of 0.1 mV s^−1^ are depicted in Figure 6. Obviously, three pairs of reduction and oxidation peaks are observed in all the both samples. Meanwhile, according to the nickel-rich material phase transition process, caused by phase transitions of hexagonal phase to monoclinic phase (H1 to M), monoclinic phase to hexagonal phase (M to H2) and hexagonal phase to hexagonal phase (H2 to H3) during the charge-discharge process(Li et al., 1993; Xie et al., 2015). The first cycle are significantly different from those for the following cycles, because of the irreversible electrochemical reaction during the first discharge cycle. However, no obvious alteration is observed between the second cycle and the third cycle, suggesting the good reversibility of lithium insertion and extraction reactions. As can be seen from the Figures 6a,b, the peak position of PL-2 material shifted is smaller than LNCA. This illustrates that the PPy materials possess lower activation energy and higher electrical conductivity for the active materials. Therefore, PPy coated layer is beneficial to reducing the electrochemical polarization as well as improving the electrochemical performance.

**Figure 7:**
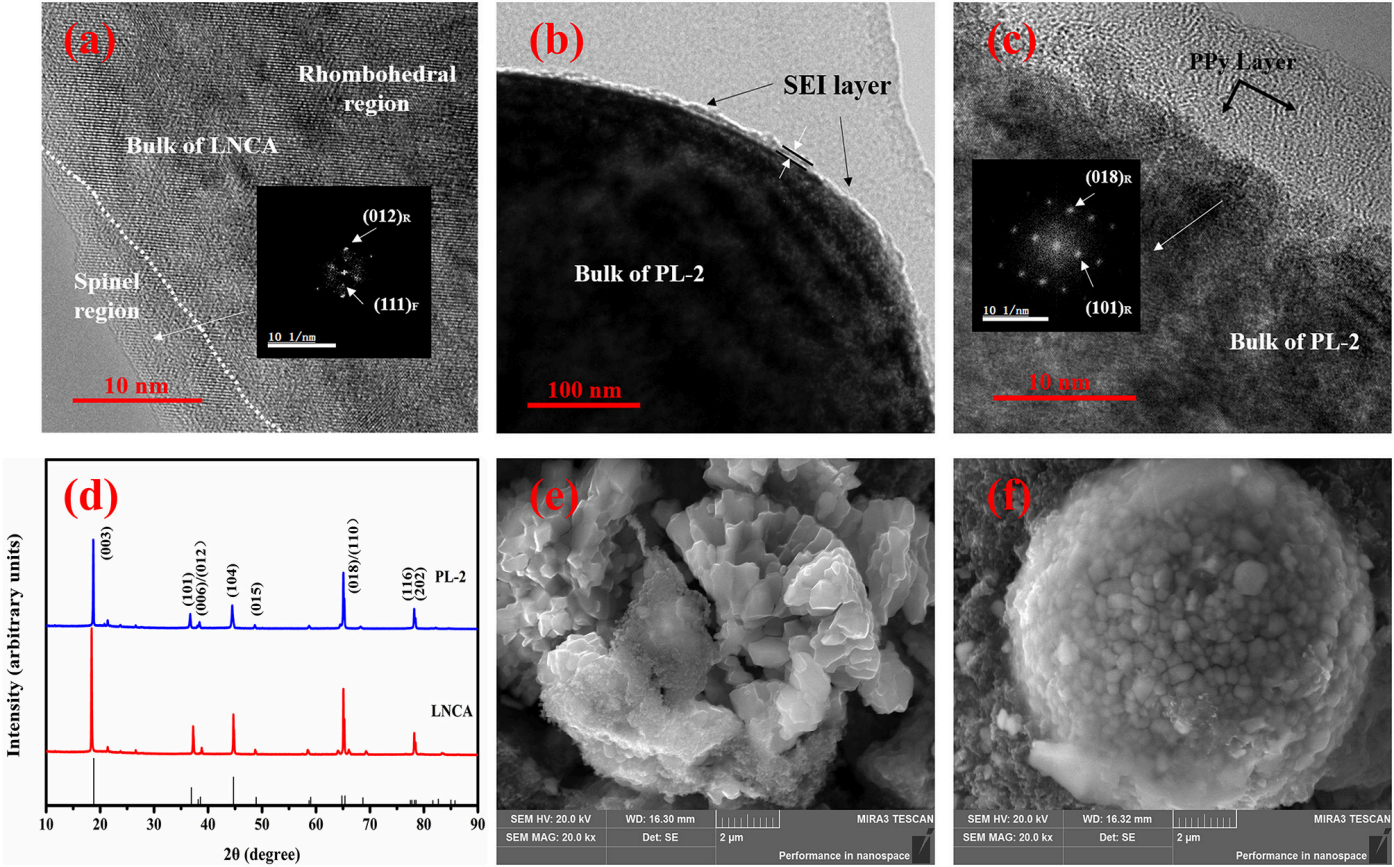
HRTEM images of: **(a)** the LNCA material after 100^th^ cycle; **(b,c)** PL-2 sample after the 100^th^ cycle; The index marked by subscript R and F is related to the rhombohedral (R-3m) phase and cubic (Fd-3m) phase. XRD pattern and SEM images of pristine LNCA and PL-2 cathode material during the charge and discharge cycles. XRD pattern of: **(d)** the pristine LNCA and PL-2 cathode material after 100^th^ cycle; SEM images of: **(e)** the LNCA material after 100^th^ cycle; **(f)** PL-2 sample after the 100^th^ cycle.

In order to study the structural evolution of bare LNCA and PL-2 samples upon cycling, HRTEM and FFT imaging is performed. Figure 7a present the HRTEM images of the bare LNCA sample after the 100 th cycle, while Figures 7b,c present the HRTEM images of the PL-2 sample after the 100 th cycle, As can be seen from Figure 7a, the distortion region is observed over distances of *ca*. 10 nm from the surface of the LNCA material after 100 th cycle. The FFT in the inset of Figure 7a exhibits a spinel-like phase (space group Fd-3m). The formation of spinel-like phase in the surface region of the cycled LNCA sample is attributed to the migration of TM ions to the Li sites without breaking down the lattice.

Figures 7b,c show the HRTEM and corresponding FFT results of the PL-2 sample. We have found that the surface region of the cycled PL-2 sample still remains in the rhombohedral phase. This is also consistent with the cycling performances and electrochemical reversibility (Figures 5a, 6). This observation likely indicates that the phase transition from layered to spinel can be suppressed by this PPy coated layer.

Furthermore, the XRD pattern and SEM images of pristine LNCA and PL-2 cathode material during the charge and discharge cycles are presented in the Figures 7d–f. According to the XRD pattern, the peaks of the both samples are basically similar. It is demonstrated that the cycled PL-2 sample still remains as the rhombohedral phase. According to SEM images of pristine LNCA and PL-2 cathode material after the charge and discharge cycles, the morphology of the pristine LNCA particles has obvious collapse, resulting in decrease of capacity in process of charge and discharge. However, the PL-2 cathode material still remains the spherical morphology after cycles. This likely indicates that the PPy maintains the structural stability of cathode material during the cycles.

## Conclusion

In summary, we have successfully synthesized a PPy-coated LNCA cathode material via Liquid-phase chemical oxidation polymerization method. According to the results of XRD, HRTEM, and FTIR, PPy was uniformly dispersed on the surface of the LNCA cathode material without changing its structure. Furthermore, the PPy coated LNCA cathode material shows excellent rate capability and capacity retention at 1, 3, and 5 C rates, indicating a good kinetics characteristic and the effectiveness of surface coating for protecting cathode materials from the side reaction between electrode and electrolyte. Meanwhile, the PPy-coated LNCA showed an improved coulombic efficiency compared with the pristine one. Therefore, as a highly Li-ions and electrons conductive material, the PPy coating shows great potential in surface modification and is expected to improve performance for other types cathode materials of LIBs.

## Author Contributions

ZC and KC conceived and designed the study; KC and ZC prepared all materials. KC and HZ conducted SEM experiments. XG and QL conducted XRD, FTIR experiments. ZC and KC analyzed the data. KC wrote the manuscript and LL, JD commented on it. ZC supervised the implementation of the project. All authors read and approved the final manuscript.

### Conflict of Interest Statement

The authors declare that the research was conducted in the absence of any commercial or financial relationships that could be construed as a potential conflict of interest.

## Supplementary Material

XRD pattern and electrochemical performance of samples in different PPy amounts (1wt%, 2wt%, 5wt%). XRD patterns of the bare LNCA, PPy coated LNCA material: PL-1, PL-2, and PL-5 samples. The initial charge/discharge profiles at 0.1 C rate of pristine LNCA cathode materials and PPy coated LNCA material: PL-1, PL-2, and PL-5 samples. The rate performance profiles and cycling performance profiles at 1 C rate of pristine LNCA cathode materials and PPy coated LNCA material: PL-1, PL-2, and PL-5 samples.
