# Supplementary material for: Improved Electrochemical Performance of Surface Coated LiNi0.80Co0.15Al0.05O2 With Polypyrrole
Source: Front Chem. 2019 Jan 9;6:648. doi: 10.3389/fchem.2018.00648 (PMC6334193; doi:10.3389/fchem.2018.00648)
Supplement: Supplementary file 1 [file Data_Sheet_1.PDF]

Supplementary Material

Improved the electrochemical performance of Polypyrrole surface coated $LiNi_{0.80}Co_{0.15}Al_{0.05}O_2$ cathode materials

Zhaoyong Chen, Kaifeng Cao, Huali Zhu, Xiaolong Gong, Qiming Liu, Junfei Duan, Lingjun Li*

AUTHOR ADDRESS

College of Materials Science and Engineering, Changsha University of Science and Technology, Changsha 410004, P. R. China.

CORRESPONDING AUTHOR

*Zhaoyong Chen, E-mail: chenzhaoyongcioc@126.com

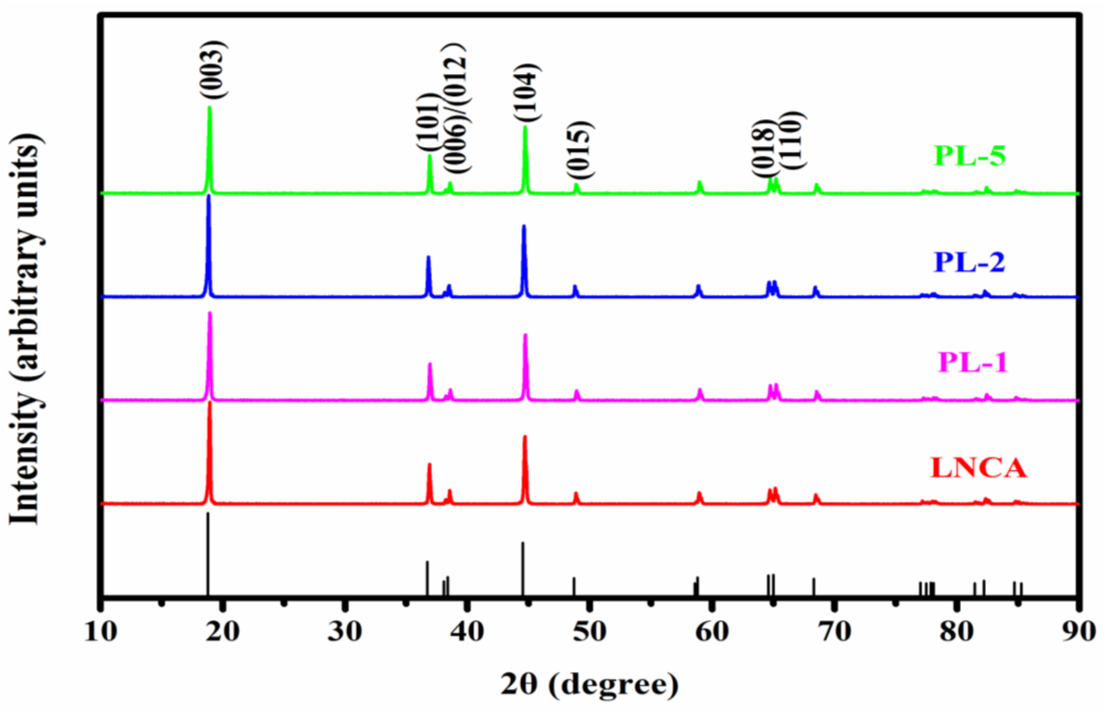

Supplementary Figure 1. XRD patterns of the bare LNCA, PPy coated LNCA material: PL-1, PL-2 and PL-5 samples.

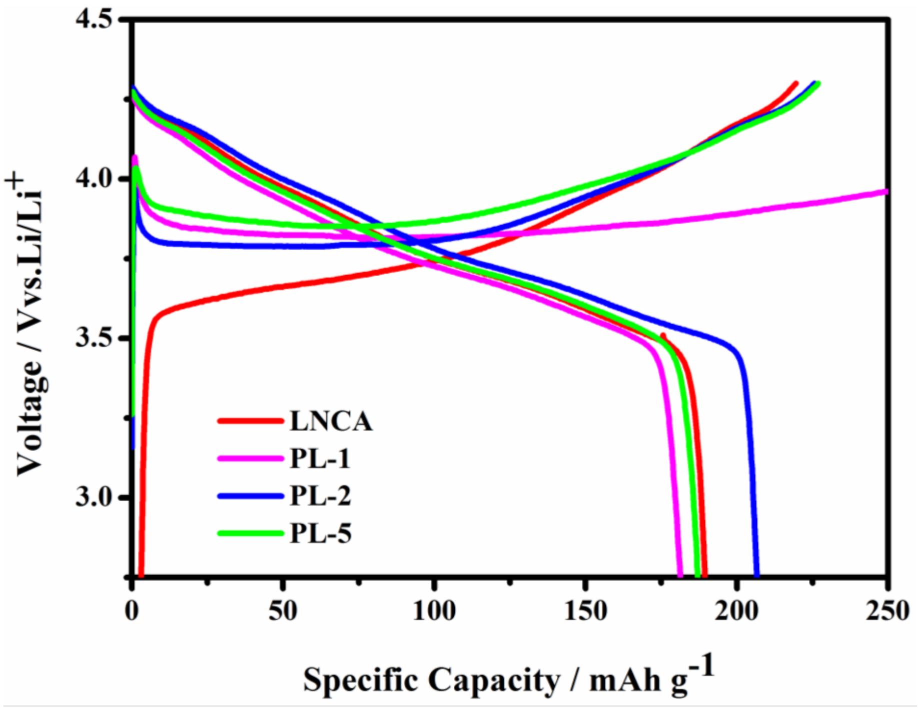

Supplementary Figure 2. The initial charge/discharge profiles at 0.1 C rate of pristine LNCA cathode materials and PPy coated LNCA material: PL-1, PL-2 and PL-5 samples.

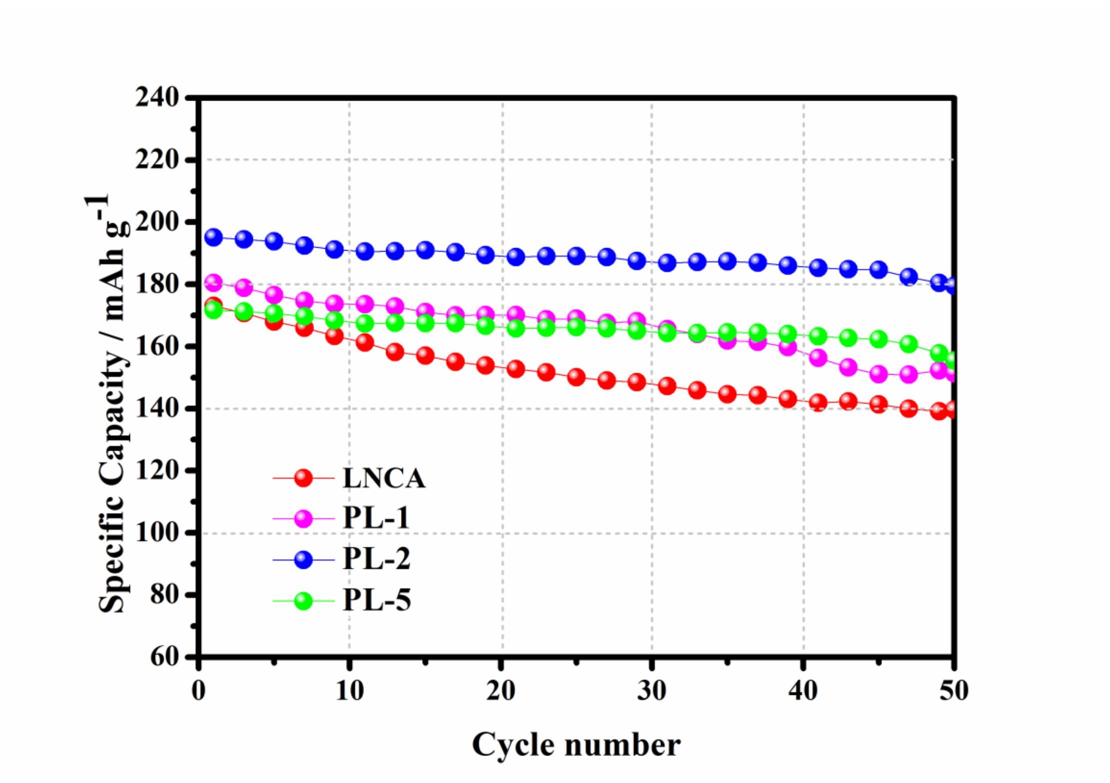

Supplementary Figure 3. The cycling performance profiles at 1 C rate of pristine LNCA cathode materials and PPy coated LNCA material: PL-1, PL-2 and PL-5 samples.

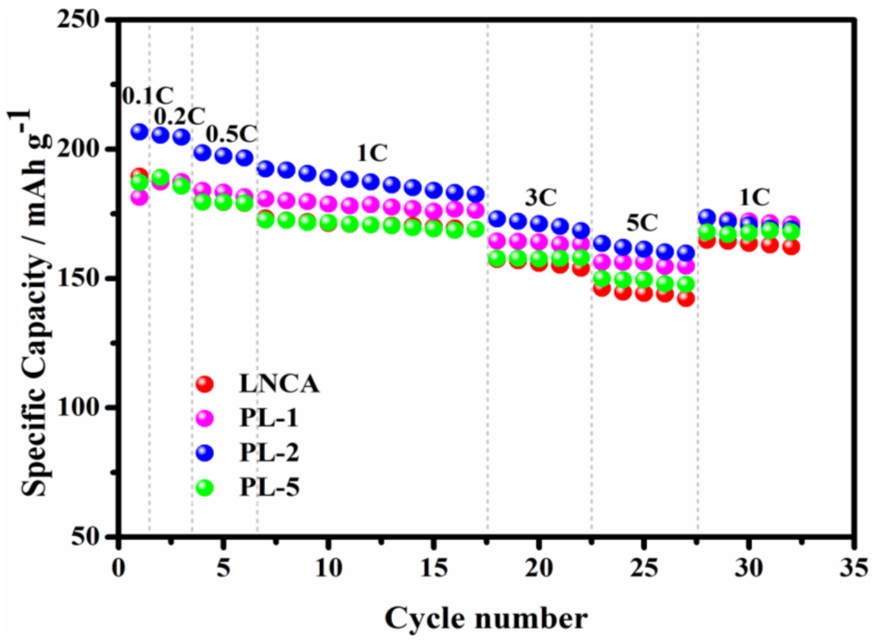

Supplementary Figure 4. The rate performance profiles of pristine LNCA cathode materials and PPy coated LNCA material: PL-1, PL-2 and PL-5 samples.
